# Supplementary material for: Trials in developing a nanoscale material for extravascular contrast-enhanced ultrasound targeting hepatocellular carcinoma
Source: PeerJ. 2020 Dec 7;8:e10403. doi: 10.7717/peerj.10403 (PMC7727372; doi:10.7717/peerj.10403)
Supplement: Supplemental Information 7 — Images obtained during this study. [file peerj-08-10403-s007.doc]

Yuuii


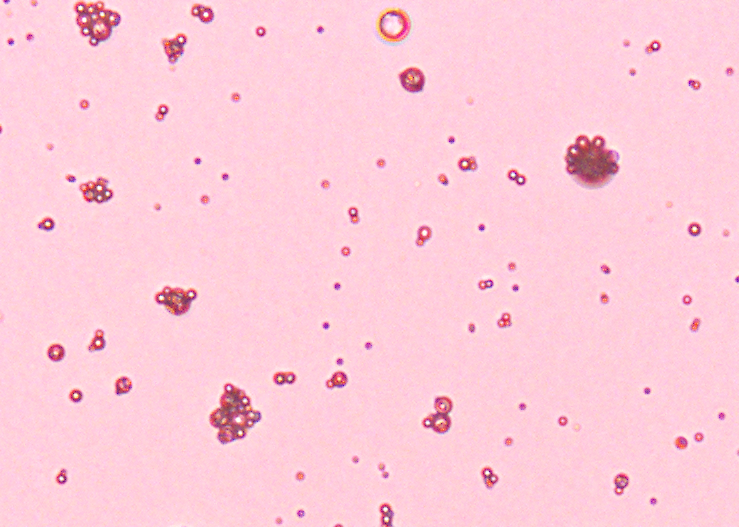


Figure Image of the LSPMbs obtained by Light microscope. The LSPMbs present round and ovoid shaped.10×40


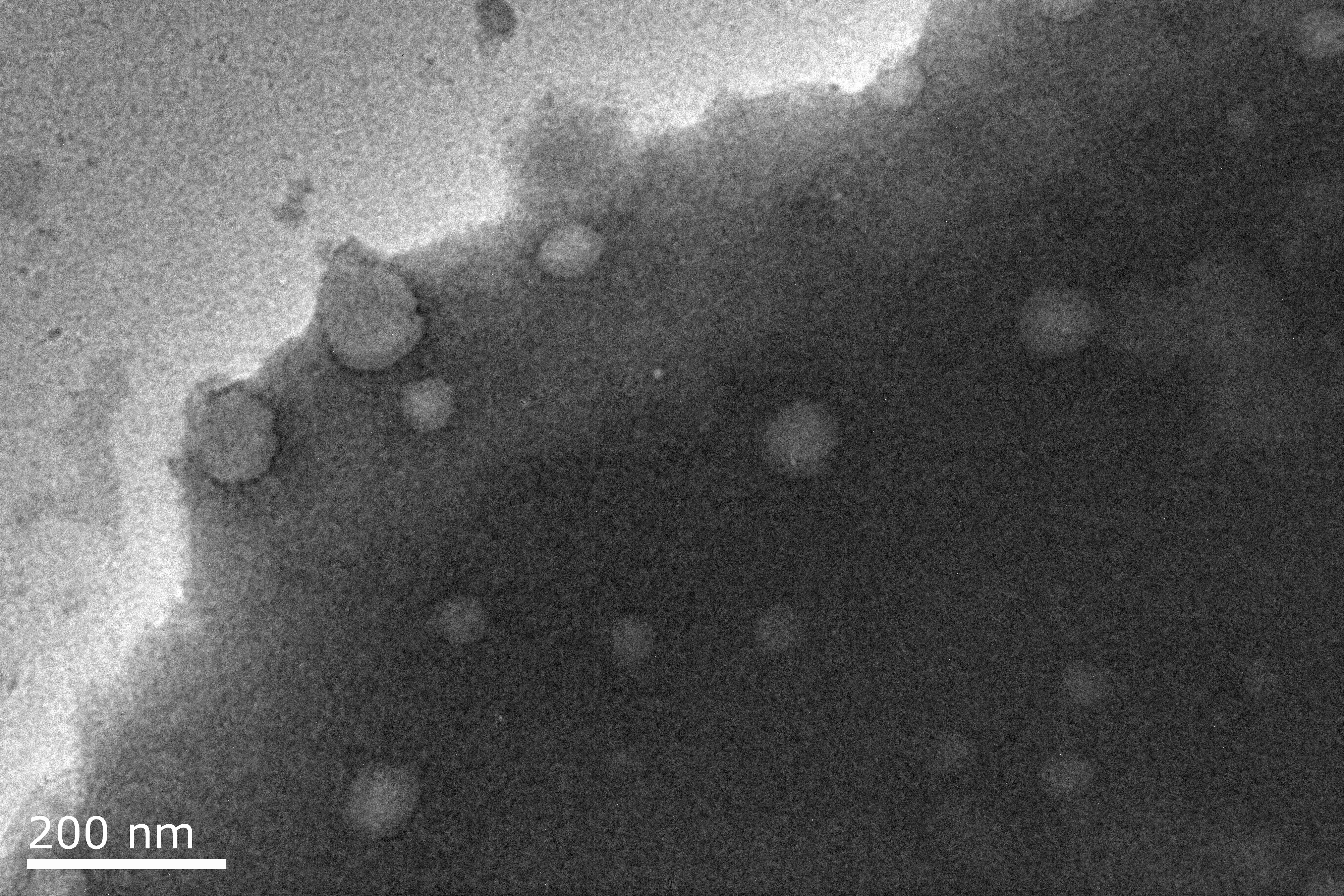


Figure Image of the LSPMbs obtained by transmission electron microscope. The LSPMbs present round shaped on a sectional view, with different size, without aggregation.


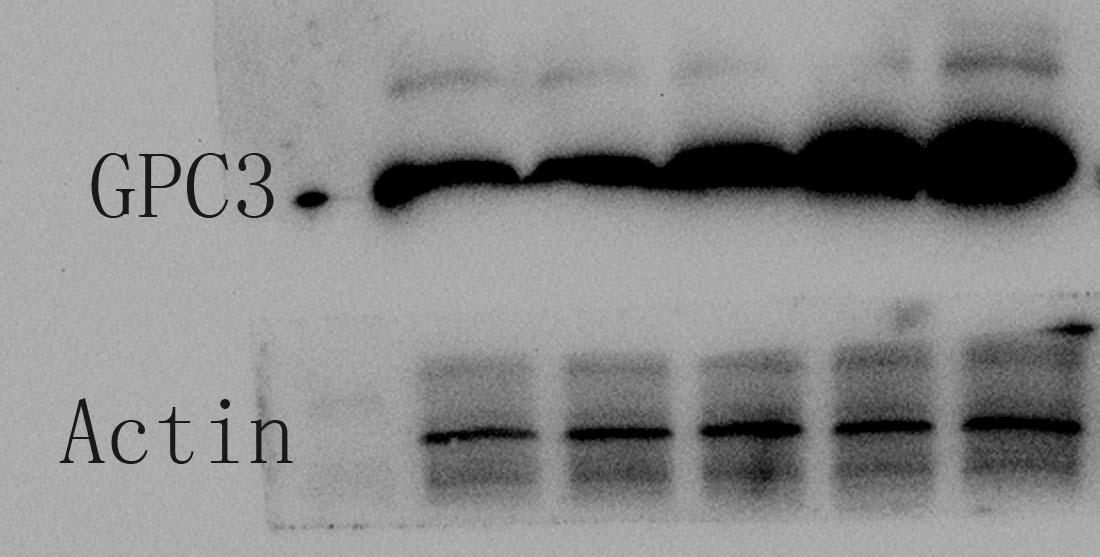


Figure 3 GPC3 is expressed in Huh-7 cells obtained by Western Blot.
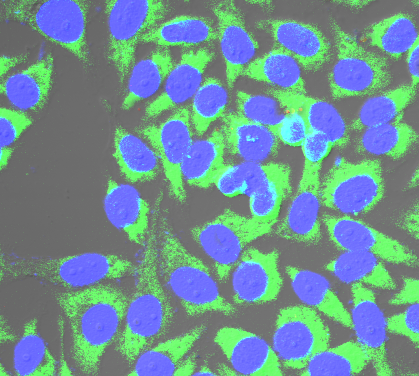


Figure Image of GPC3 expression of Huh-7 cells obtained by confocal laser scanning microscope.


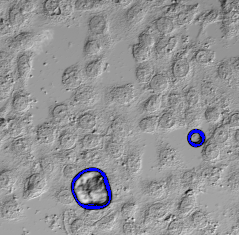

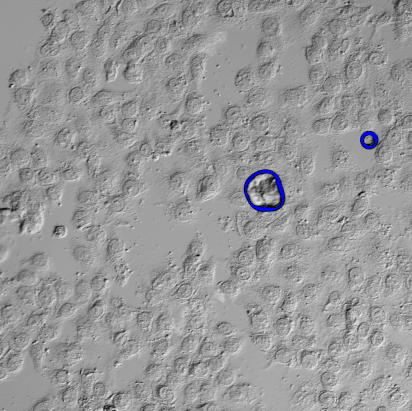

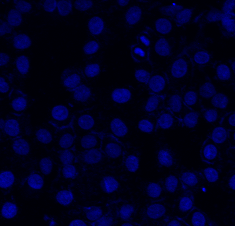

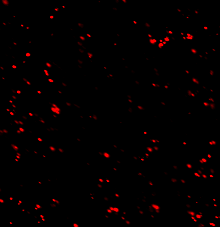

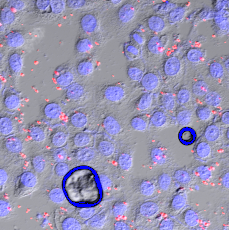


Figure Images of Huh-7 cells incubated with DiI lablled LSPMbs obtained by light microscope and confocal laser scanning microscope. DiI lablled LSPMbs present read, and cell nucleus of Huh-7 cells presents blue after DAPI staining.


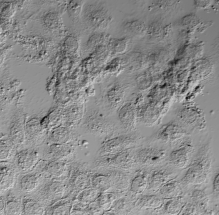

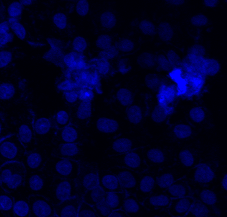

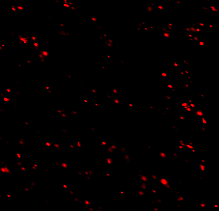

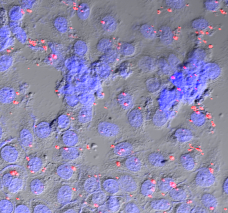


Figure Images of Huh-7 cells incubated with DiI lablled LSGMbs obtained by light microscope and confocal laser scanning microscope. DiI lablled LSPMbs present read, and cell nucleus of Huh-7 cells presents blue after DAPI staining.


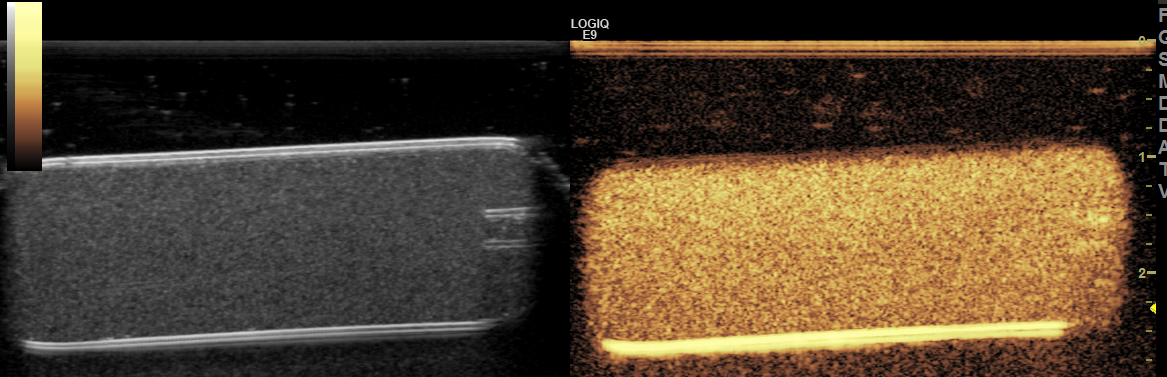


Figures Image obtained from LSPMbs (1.5mg/mL) in a tube scanned by ultrasound with a scanner of frequency of 12 MHz (MI 0.6).


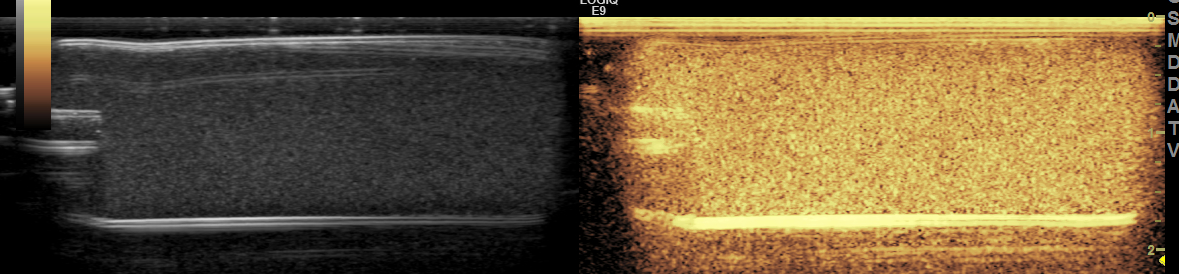


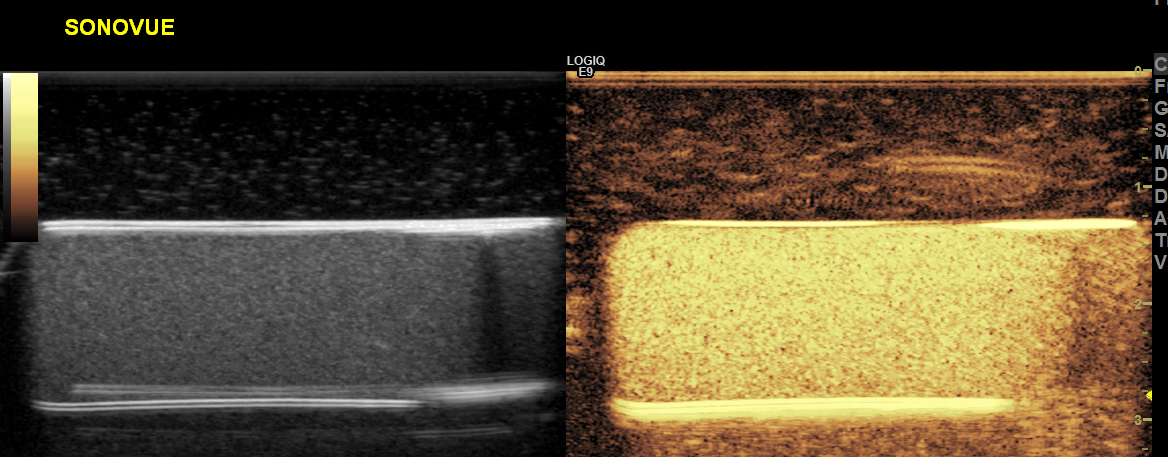


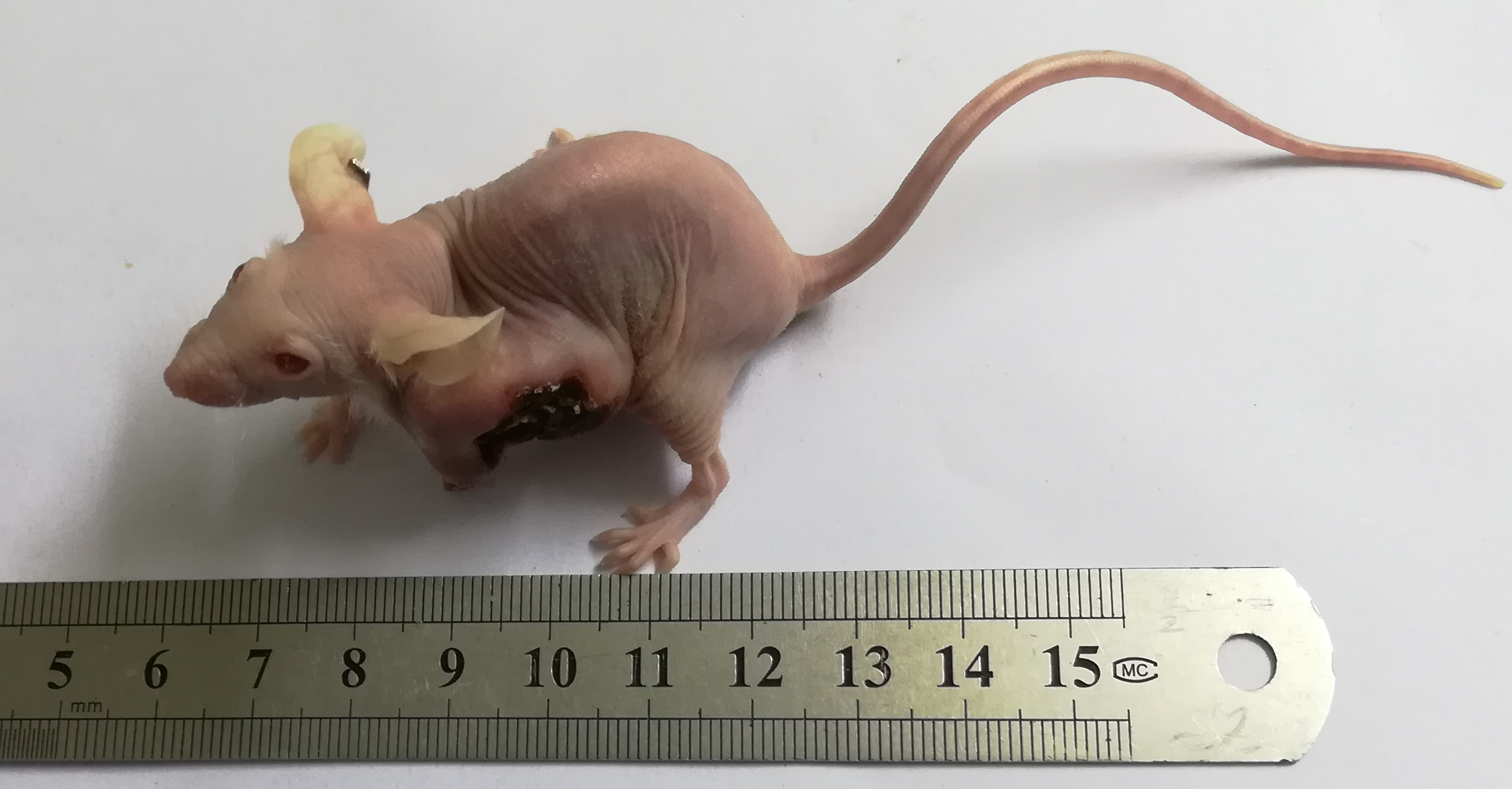


Figure A mouse model with xenograft tumor.


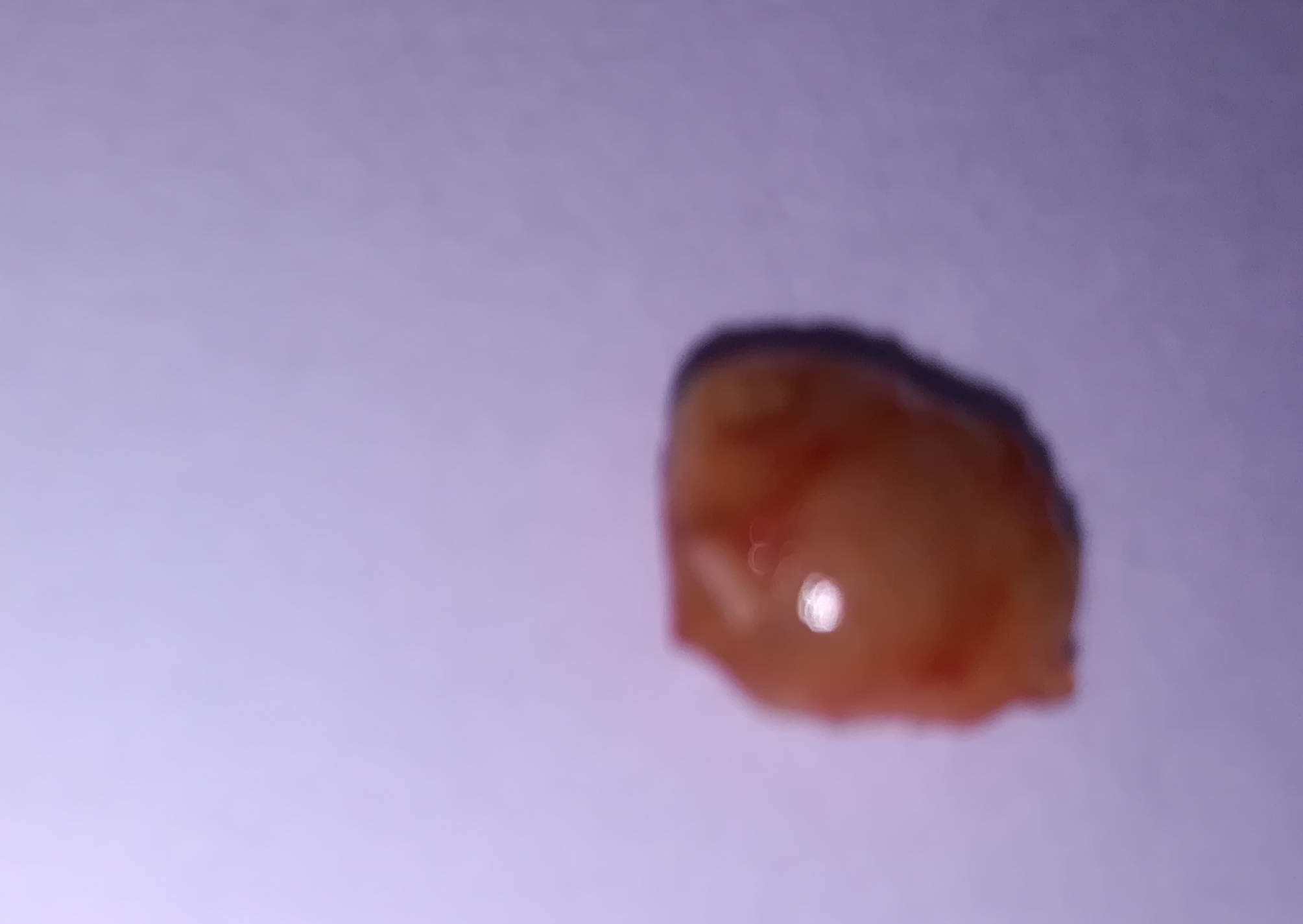


Figure


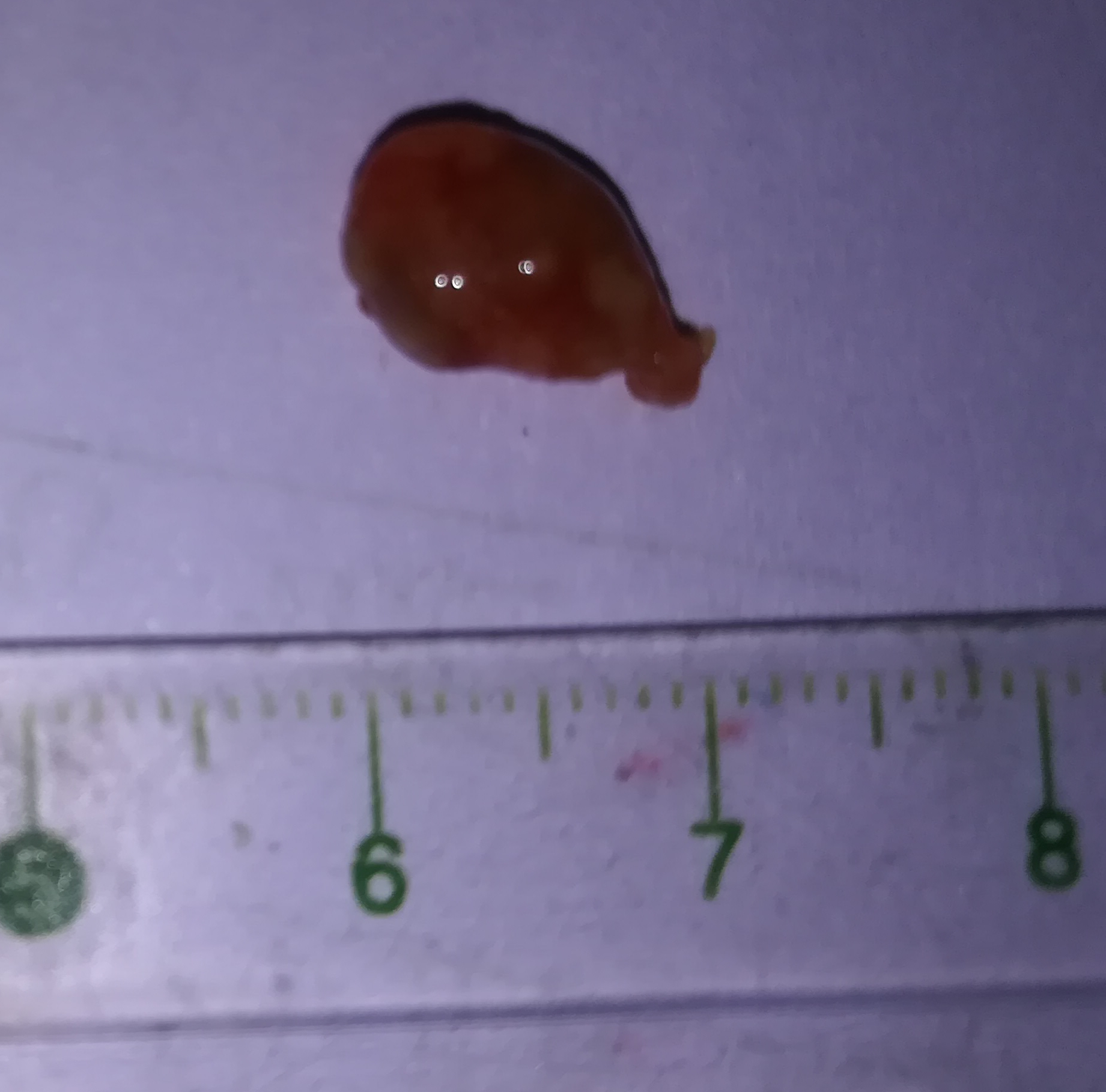


Figure A sample of xenograft tumor of the mouse.


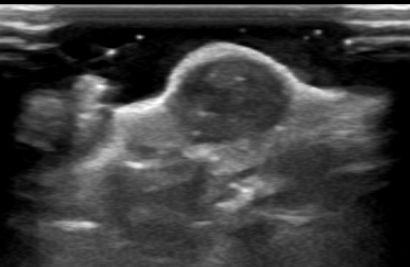


Figure Image of xenograft tumor of the mouse obtained by ultrasound with a scanner of frequency of 12 MHz ,


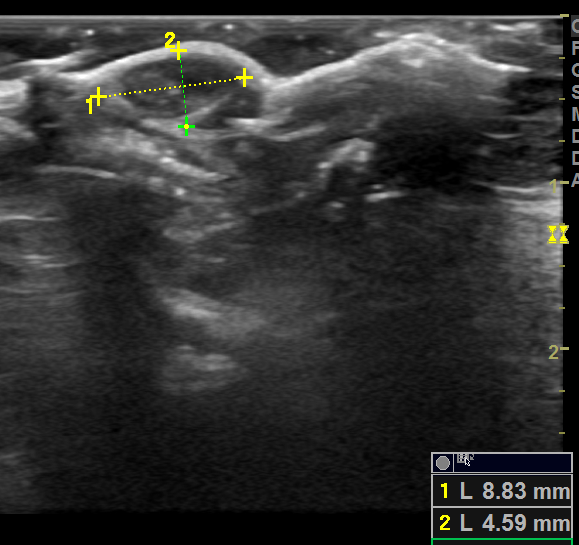


Figure


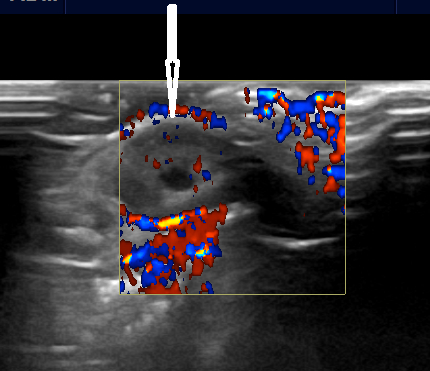


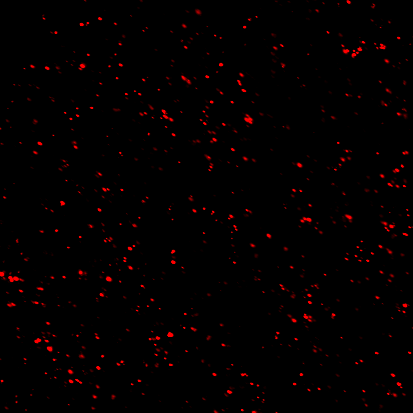

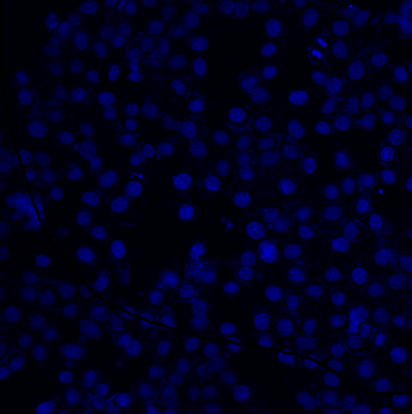

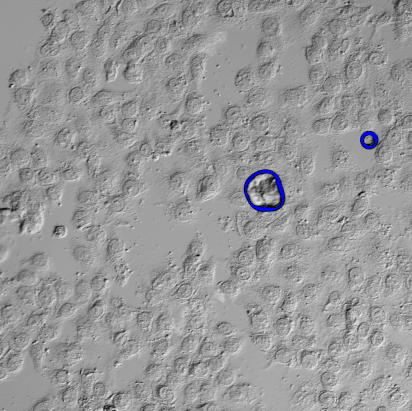

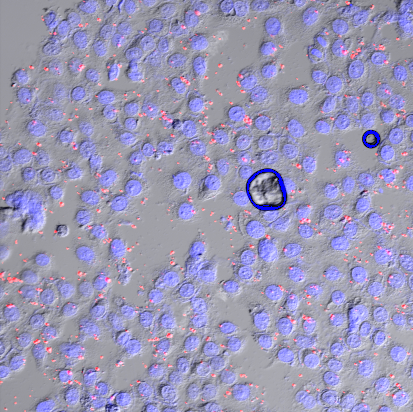
 Figure


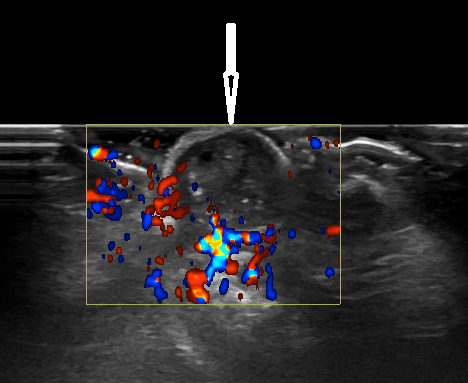


Figure Image of xenograft tumor of the mouse obtained by duplex ultrasound with a scanner of frequency of 12 MHz


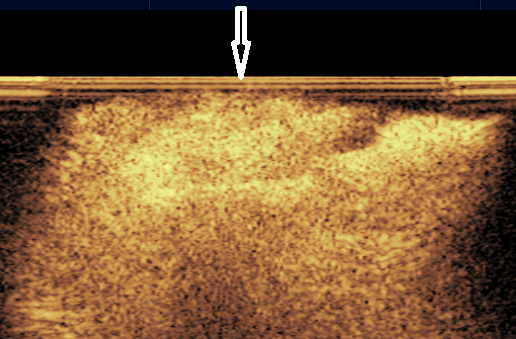


Figure Image of xenograft tumor of the mouse obtained by ultrasound with a scanner of frequency of 12 MHz (MI 0.6) after intravenously injection of 0.2 mL LPSMbs.


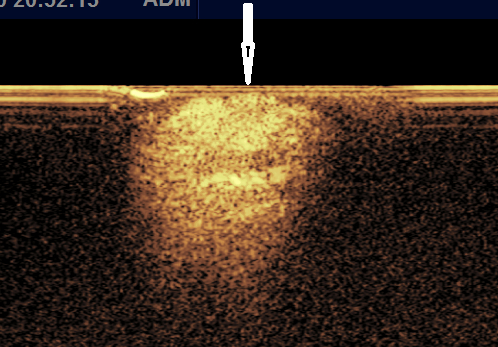


Figure


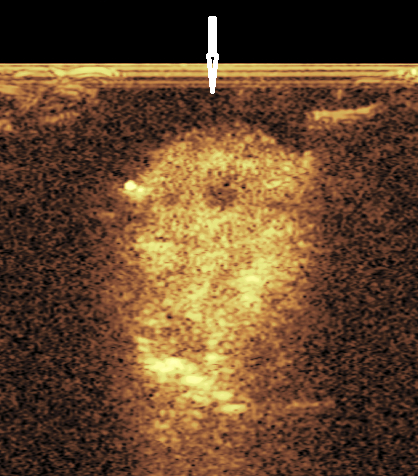


Figure


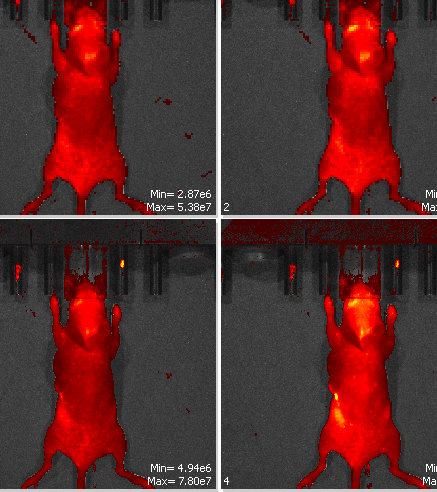


Figure Images obtained by an IVIS Lumina image system (Xenogen) (IVIS® Lumina XR) (Caliper life sciences) after intravenously injection of 0.2 mL Cy5.5 labelled LPSMbs.
